# Influences of population pressure change on vegetation greenness in China's mountainous areas

**DOI:** 10.1002/ece3.3424

**Published:** 2017-09-25

**Authors:** Wei Li, Xiubin Li, Minghong Tan, Yahui Wang

**Affiliations:** ^1^ Key Laboratory of Land Surface Pattern and Simulation Institute of Geographical Sciences and Natural Resources Research Chinese Academy of Sciences Beijing China; ^2^ College of Resources and Environment University of Chinese Academy of Sciences Beijing China; ^3^ International College University of Chinese Academy of Sciences Beijing China

**Keywords:** Chinese mountainous areas, land‐use intensity, migration, Normalized Difference Vegetation Index, population pressure, regional variation, vegetation greenness

## Abstract

Mountainous areas in China account for two‐thirds of the total land area. Due to rapid urbanization, rural population emigration in China's mountainous areas is very significant. This raises the question to which degree such population emigration influences the vegetation greenness in these areas. In this study, 9,753 sample areas (each sample measured about 64 square kilometers) were randomly selected, and the influences of population emigration (population pressure change) on vegetation greenness during 2000–2010 were quantitatively expressed by the multivariate linear regression (MLR) model, using census data under the condition of controlling the natural elements such as climatic and landform factors. The results indicate that the vegetation index in the past 10 years has presented an increasing overall trend, albeit with local decrease in some regions. The combined area of the regions with improved vegetation accounted for 81.7% of the total mountainous areas in China. From 2000 to 2010, the rural population significantly decreased, with most significant decreases in the northern and central areas (17.2% and 16.8%, respectively). In China's mountainous areas and in most of the subregions, population emigration has significant impacts on vegetation change. In different subregions, population decrease differently influenced vegetation greenness, and the marginal effect of population decrease on vegetation change presented obvious differences from north to south. In the southwest, on the premise of controlling other factors, a population decrease by one unit could increase the slope of vegetation change by 16.4%; in contrast, in the southeastern, northern, northeastern, and central area, the proportion was about 15.5%, 10.6%, 9.7%, and 7.5%, respectively, for improving the trend of NDVI variation.

## 1 INTRODUCTION

At present, more than half of the world's population lives in cities. According to the prediction of the United Nations Population Division, urban population will increase to 5 billion by 2030, mainly concentrated in Africa and Asia (UNFPA, 2014). China is the largest developing country in the world, and since the 21st century, about 15 million people are moving from rural areas to cities every year (Cai, Wang, & Yang, 2007). Accordingly, the rural population in China decreased by about 17% during the first 10 years of the 21st century (Li, Sun, Tan, & Li, 2016). This entails dramatic changes in the spatial distribution of the rural population (Cai, Yang, Wang, & Xiao, 2014; Liu, Pan, Zhu, & Li, 2015; Liu, Zhao, et al., 2015; Zhang, Peng, & Mao, 2006).

In mountainous areas, population emigration is likely to reduce the intensity of human activities in out‐migrating places, and consequently, to promote vegetation greenness. On one hand, a reduction of agricultural labor force can significantly reduce firewood consumption of mountain farmers (He, Yan, Zhou, & Li, 2016). On the other hand, population emigration reduces the cultivated land‐use rate in mountainous areas (Bradley, 2016). Previous studies (Aide & Grau, 2004; Aide et al., 2013; Chazdon, 2008; Jennifer, Tobias, & Radeloff, 2012; Li & Li, 2016) have found that decreasing firewood cutting and accelerating the abandonment of cultivated land are conducive to the restoration of ecosystems and, consequently, the protection of biodiversity.

At present, due to the dual influences of natural and human factors on vegetation change, it is difficult to quantitatively identify the contribution ratio of human activities, especially population migration, to vegetation greenness change. According to the existing research, studies on the factors influencing vegetation change can be divided into two aspects, natural factors (primarily temperature and precipitation) (Chen et al., 2015; Duo, Zhao, Qu, Jing, &Xiong, 2016 Gessner et al., 2013; He, Guo, Dixon, & Wilmshurst, 2012; Liu & Lei, 2015; Liu, Pan, Zhu, & Li 2015; Liu, Zhao, et al., 2015; Piao et al., 2012; Sun, Yang, Zhang, & Wang, 2015) and human activities (Aide & Grau, 2004; Cao, Ma, Yuan, & Wang, 2014; Feng, Ma, Jiang, Wang, & Cao, 2015; Gartzia, Pérez‐Cabello, Bueno, & Alados, 2016; Li, Wu, & Huang, 2012; Lu et al., 2015; Sun et al., 2015; Tousignant, Pellerin, & Brisson, 2010). The natural factors have received extensive attention. It is proved that the impact of climate factors is crucial on vegetation growth in some regions (Bao et al., 2015; Chuai, Huang, Wang, & Bao, 2013; Piao, Mohammat, Fang, Cai, & Feng, 2006). Especially, temperature is claimed to play a dominated role compared to precipitation (Thavorntam & Tantemsapya, 2013; Tian et al., 2015). Some scholars have explored the relationship between human activities and vegetation change through the correlation analysis method (Cai et al., 2014; Lu et al., 2015). For example, Cai et al. (2014) have explored the relationship between population emigration and vegetation change at the county scale in the karst areas of southwest China, using spearman correlation analysis, and found a positive influence of population emigration on vegetation index. However, they only used one single factor and did not control other variables. Lu et al. (2015), using Pearson's correlation analysis, selected a large number of factors (such as population, labor force, GDP, investment.) at the provincial scale as explanatory variables and analyzed the influences of China's social and economic factors on vegetation index, without controlling the natural factors. Other authors have tried to isolate the influence of human activity from the comprehensive influences using residual analysis (Evans & Geerken, 2004; Ferrara, Salvati, Sateriano, & Nolè, 2012; Sun et al., 2015; Tousignant et al., 2010; Wang, Wang, Zhang, & Zhang, 2015; Wessels et al., 2007). For example, Wessels et al. (2007) have used residual analysis to isolate the influences of human activities on vegetation productivity in a study on the impacts of land degradation in South Africa. In mountainous areas, Wang et al. (2015) have investigated the influences of climate and human activity factors on the vegetation of southern China using residual analysis, obtaining the regression equations of the vegetation index for temperature and precipitation, whereas the influences of human factors were completely explained by the residual terms of the regression equation. However, the residual analysis method could only reveal the positive or negative effects of human activity instead of identifying the types, intensity, and contribution ratio of human activities.

Such studies have deepened our understanding of the relationship between human activities and vegetation change. However, these approaches could not identify the types, intensity, and contribution ratios of human activities that influence vegetation greenness change and do not exclude the influences of natural factors, such as temperature and precipitation. In addition, these studies always choose a certain administrative unit as the research object. However, due to the large land area and complex topography of mountainous areas, the spatial differences inside the administrative unit are significant and therefore using an administrative unit as the analysis unit increases the uncertainty of the results.

Mountainous areas in China account for two‐thirds of the total land area; they are characterized by high intensity of human activity and fragile ecological environments. Furthermore, a number of large rivers rise in China's mountainous areas, such as the Yangtze River, the Yellow River, and the Lancang River, as well as some international rivers; the state of the ecological vegetation of these areas significantly affects the hydrological conditions of these rivers in China as well as in neighboring countries. So, it is very important to evaluate the effects of human activities on mountainous vegetation in China. This study chose the widely used vegetation index NDVI (Normalized Difference Vegetation Index) as an indicator of vegetation conditions (Fu & Burgher, 2015; Guay et al., 2014; He et al., 2012; Liu & Gong, 2012; Starns, Weckerly, Ricca, & Duarte, 2015; Stow et al., 2003; Zhang, Zhang, Dong, & Xiao, 2013). Furthermore, we used two indices, population pressure (population density) and land‐use intensity, to express human activity intensity. Then, 9,753 samples of 64 square kilometers each were selected, to reduce the uncertainty caused using administrative units with a large area. This allowed us to quantitatively assess the effects of human activities on the vegetation conditions, using the multivariate linear regression (MLR) model under the condition of controlling the influences of natural factors.

## 2 MATERIALS AND METHODS

### 2.1 Study area and data source

**Figure 1:**
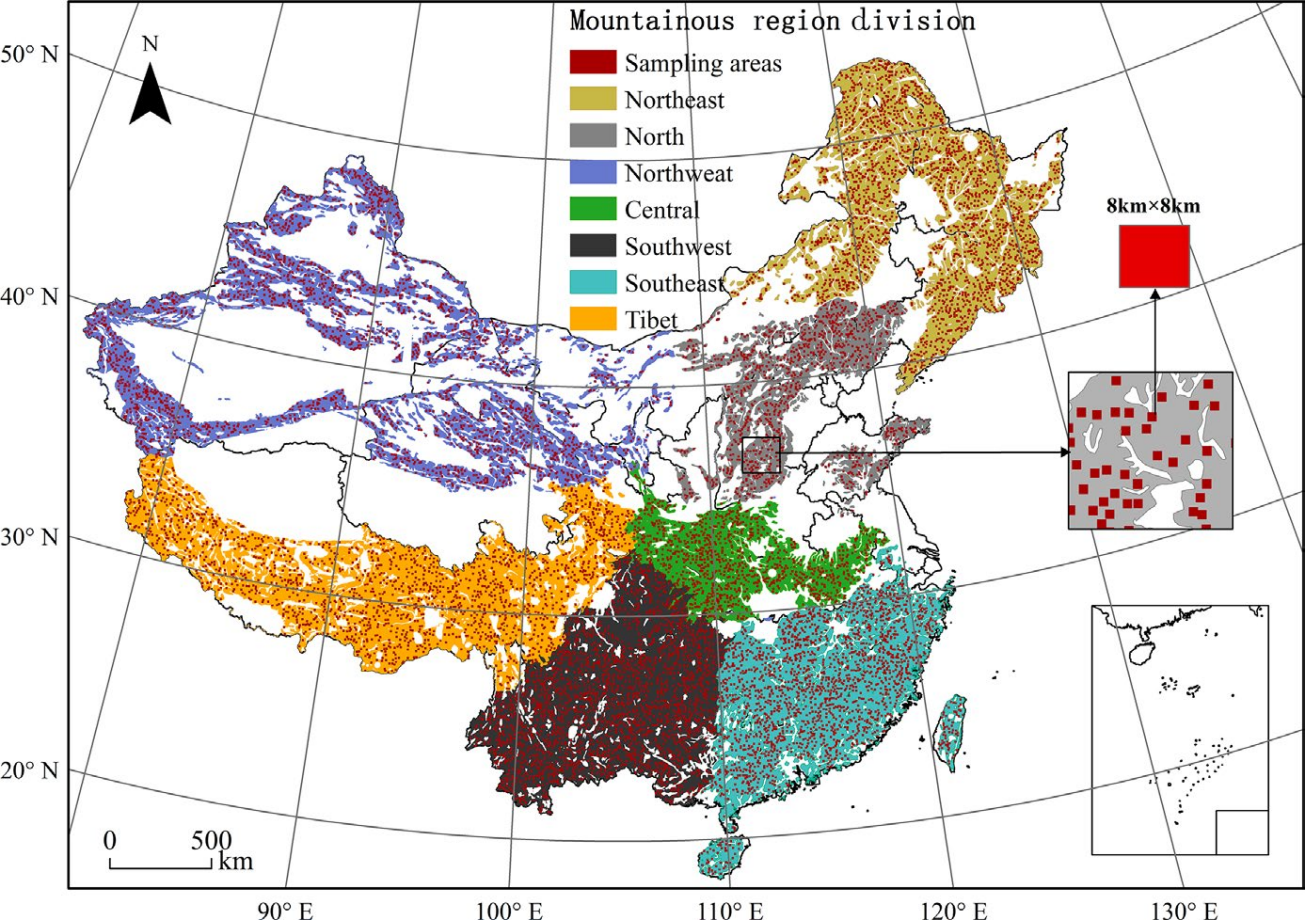
Regional division and sample spatial distribution of China's mountainous areas. NOTE: the landform map is derived from State Key Laboratory of Resources and Environmental Information System, Institute of Geographic Sciences and Natural Resources Research, Chinese Academy of Sciences; measuring scale is 1:4,000,000

The study area covers about five million square kilometers (Figure 1). According to previous research (Guo & Zhang, 1986), China's mountainous areas can be divided into seven regions: northwest, northeast, north, central, southeast, southwest, and Tibet.

In this study, we mainly used the following data: NDVI data, surface meteorological data, SRTMDEM (Shuttle Radar Topography Mission, Digital Elevation Model) data, land‐use data, and population density data. The synthetic products MODND1M of the NDVI MODIS during the period 2000–2010 were provided by the International Scientific & Technical Data Mirror Site, Computer Network Information Center, Chinese Academy of Sciences (GSCLOUD), with a spatial resolution of 1 × 1 km and a temporal resolution of 1 month. In northern China, the growing season is from April to October (Li, Sun, Tan, & Li, 2016); therefore, the average NDVI value for the growing season was adopted to replace the annual average NDVI value in this study. Temperature and precipitation data from 2000 to 2010 were obtained from the China Meteorological Data‐Sharing Service System (“Climatic Data Center, National Meteorological Information Center, China Meteorological Administration”), with a spatial resolution of 0.5 × 0.5° and a temporal resolution of 1 month. The SRTM DEM data were obtained from GSCLOUD, with a spatial resolution of 90 × 90 m. According to the DEM data, the gradient map and aspect map, with a spatial resolution of 90 × 90 m, can be obtained by the spatial analysis module of the software ArcGIS10.2. Land‐use data of 2000 and 2010 (Xu et al, 2015), with a spatial resolution of 100 × 100 m, were obtained from Global Change Research Data Publishing & Repositor, Institute of Geographic Sciences and Natural Resources Research, Chinese Academy of Sciences. In this data set, there are seven main land use types. These are arable land, woodland, grassland, water area, construction land, unused land, and other areas. Population density data mainly refer to the research report of Tan, Li, Li, and Li (2016), and the spatial distribution diagram of population density is modeled based on nighttime light image data, land‐use data and the fifth and sixth nationwide census data.

**Figure 2:**
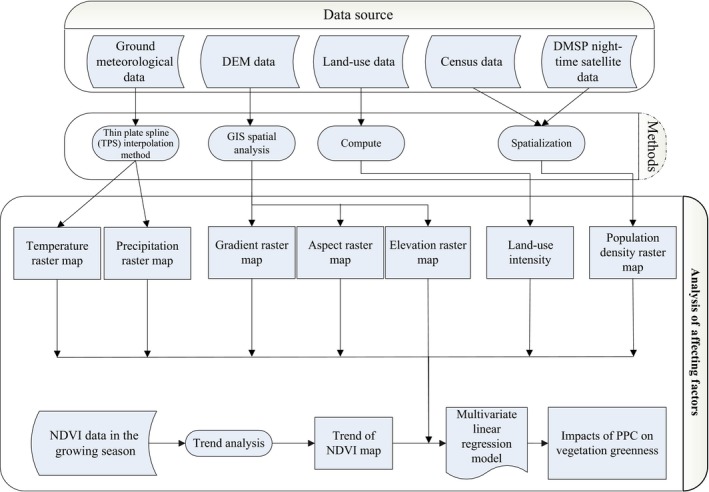
Flowchart of the algorithm used to estimate the factors affecting vegetation greenness

Figure 2 shows the overall design of this study, expressed as a flowchart.

## 3 METHODS

### 3.1 Interpolation of meteorological data

Original temperature and precipitation data were grid data sets with a precision of 0.5 × 0.5°. These datasets were obtained by interpolation based on 2,472 weather stations across the country. In this study, on the platform of the ANUSPLIN (The Australian National University, Canberra, ACT, Australia) software, we carried out an interpolation on the grid data, using elevation as the covariate via thin‐plate spline interpolation (Liu et al., 2008; Qian, Lu, & Zhang, 2010), thereby obtaining grid data with an accuracy of 1 × 1 km.

### 3.2 Trend analysis of NDVI and meteorological data

As the interannual variabilities of temperature, precipitation, and NDVI were obvious, trend analysis was used to calculate their variation trends from 2000 to 2010 (Tian et al., 2015; Wang et al., 2015; Zhao et al., 2015). Here, we use temperature as an example to introduce the calculation process for this method:

(1) $$T_{\_}\mathrm{Slope}=\frac{m\sum_{j=1}^{m}jt_{j}-\sum_{j=1}^{m}j\sum_{j=1}^{m}t_{j}}{m\sum_{j=1}^{m}j^{2}-{\left(\sum_{j=1}^{m}j\right)}^{2}},$$

where *T_*Slope is the slope of the temperature change, *m* is the fixed number of years of the study, equaling 11 in this study, and *t*_*j*_ is the temperature of the *j*‐th year. When *T_*Slope < 0, the temperature presents a decreasing trend during this study period; otherwise, it presents an increasing trend. In this study, the data were processed at the pixel level, and the average value of each index was determined in the sampling scope using the zonal statistics tool of ArcGIS 10.2.

### 3.3 Calculation of land‐use intensity (LUI)

According to the comprehensive analysis methods to measure LUI proposed in previous studies (Gao, Liu, & Zhuang, 1998; Liu, 1997; Wang, Liu, & Zhang, 2001; Zhuang & Liu, 1997), the land was divided into four land use grades, that is, the unused land grade (with a grading index of 1), which contains saline‐alkali land, marsh land, sand land, bare land, and other unused or hardly used land, for instance, alpine desert and tundra; the forest–grass–water land grade (with a grading index of 2), which includes forest land, grass land, and water areas; the agricultural land grade (with a grading index of 3), which includes cultivated land, garden land, and artificial grassland; and the urban settlement land grade (with a grading index of 4), which includes town land, residential land, and industrial and traffic land. The calculation formula for the comprehensive index of LUI is as follows:

(2) $$\begin{array}{cc} & I=100\times \sum_{1}^{n}M_{i}\times S_{i}\\ & I\in \left[100,\right.\left.400\right] \end{array}$$

where *I* represents the land‐use intensity, *i* is the number of land‐use intensity grades, *M*_*i*_ refers to the grading index of the *i‐th* land‐use intensity grade, and *S*_*i*_ represents the area percentage of the *i*_th_ land‐use grade. In this study, to investigate the impacts of population pressure change on vegetation greenness in rural areas, the regions of urban land, water bodies, and nature reserves were excluded, which can eliminate the influences of low vegetation coverage rates in urban and water areas and low human activity intensities in nature reserves.

### 3.4 Selection of influencing factors

In terms of studying the influencing factors on vegetation change, this article mainly selects natural factors and human activities (Table 1). For natural factors, due to the obvious differences in the interannual change of temperature and precipitation, the variation trends of annual total precipitation and annual average temperature were used in the study period. Besides, gradient and aspect determine the vegetation site conditions and are also introduced into the model as the explanatory variables. The differences in land‐use intensity can reflect the influence degree of human land‐use activities on vegetation change (Zhuang & Liu, 1997), which can quantitatively reveal the comprehensive level of regional land use (Wang , Liu, & Zhang, 2001). Here, population density change represents the indicator that reflects population pressure.

**Table 1:** Main variables causing vegetation changes and their descriptions in mountainous areas of China

Indicator	Description	Variable	Unit
Explained variable			
Annual NDVI in the growing season	Variation in trend of NDVI for 2000–2010	Slope NDVI	–
Explanatory variable			
Population density change	Variation of population density from 2000 to 2010	Population pressure change	Inhabitants/m²
Control variables			
Land‐use intensity change	Variation of land‐use intensity from 2000 to 2010	LUIC	–
Annual total precipitation in the growing season	Variation in trend of precipitation for 2000–2010	Slope precipitation	–
Annual average temperature in the growing season	Variation in trend of temperature for 2000–2010	Slope temperature	–
Average gradient	Average gradient in a sample	Average gradient	Degree
Average aspect	Average aspect in a sample	Average aspect	–
Average elevation	Average elevation in a sample	Average elevation	Meter

### 3.5 Sample selection

For this study, the scope of mountainous areas was extracted from the geomorphological map of China. Based on the scope of mountainous areas, 11,025 samples were randomly selected by the software ArcGIS 10.2, using systemic‐random sampling with equal spatial distance between samples; each sample represented a square region with an edge length of 8 km (Figure 1). Data preprocessing was carried out on the basis of the original samples, such as to exclude overlap samples from the map and outliers, leaving 9,753 samples for analysis.

### 3.6 Establishment of multivariate linear regression (MLR) model

Using a multivariate linear regression method, we built the explanatory model for the factors influencing vegetation greenness changes. The explanatory variables include all natural factors and factors related to human activity in Table 1. The model adopted in this study was as follows:

(3) $$\text{Slope}\_\mathrm{NDVI}=\alpha \cdot \text{PPC}\;+\beta \cdot \text{LUIC}+\gamma \cdot X_{1}+\delta \cdot X_{2}+\varepsilon$$

where α, β, γ, and δ are the coefficients of the variables of the multivariate linear regression (MLR) model, respectively, ε is the error term caused by unobservable factors. To reveal the influences of population emigration on the trend of NDVI variation, close attention was paid to the variable population density change; the remaining variables were introduced into the model as control variables, of which *X*_1_ represents the climatic factors, including temperature and precipitation, and *X*_2_ represents the topographic factors, including elevation, gradient, and aspect. After model establishment, F and T tests were carried out, respectively. Table 2 presents the basic statistics and situation of research variables which introduced into the MLR model.

**Table 2:** Summary statistics of variables

Variables	Minimum	Maximum	Mean	*SD*	Unit
Explained variable					
NDVI_Slope*	−78.46	176.29	32.75	33.48	–
Explanatory variable					
PPC	−206.77	740.20	−11.17	28.22	Inhabitants/m²
Control variables					
LUIC	−33.22	37.20	−0.02	1.36	–
Slope temperature**	−83.96	165.66	28.30	43.36	–
Slope precipitation	−37.66	1,675.20	273.97	371.75	–
Average aspect	63.65	268.42	177.93	16.64	–
Average gradient	0.83	36.94	13.69	7.42	Degree
Average elevation***	2.39	8.71	6.87	1.12	–

All operations were implemented by Stata 13.0; The number of samples for the statistics is 9,753; * is 10,000 times that of NDVI_Slope, ** is 1,000 times that of Slope temperature, and *** is the natural logarithmic of original Average Elevation.

## 4 RESULTS

### 4.1 Spatial–temporal variation of NDVI

**Figure 3:**
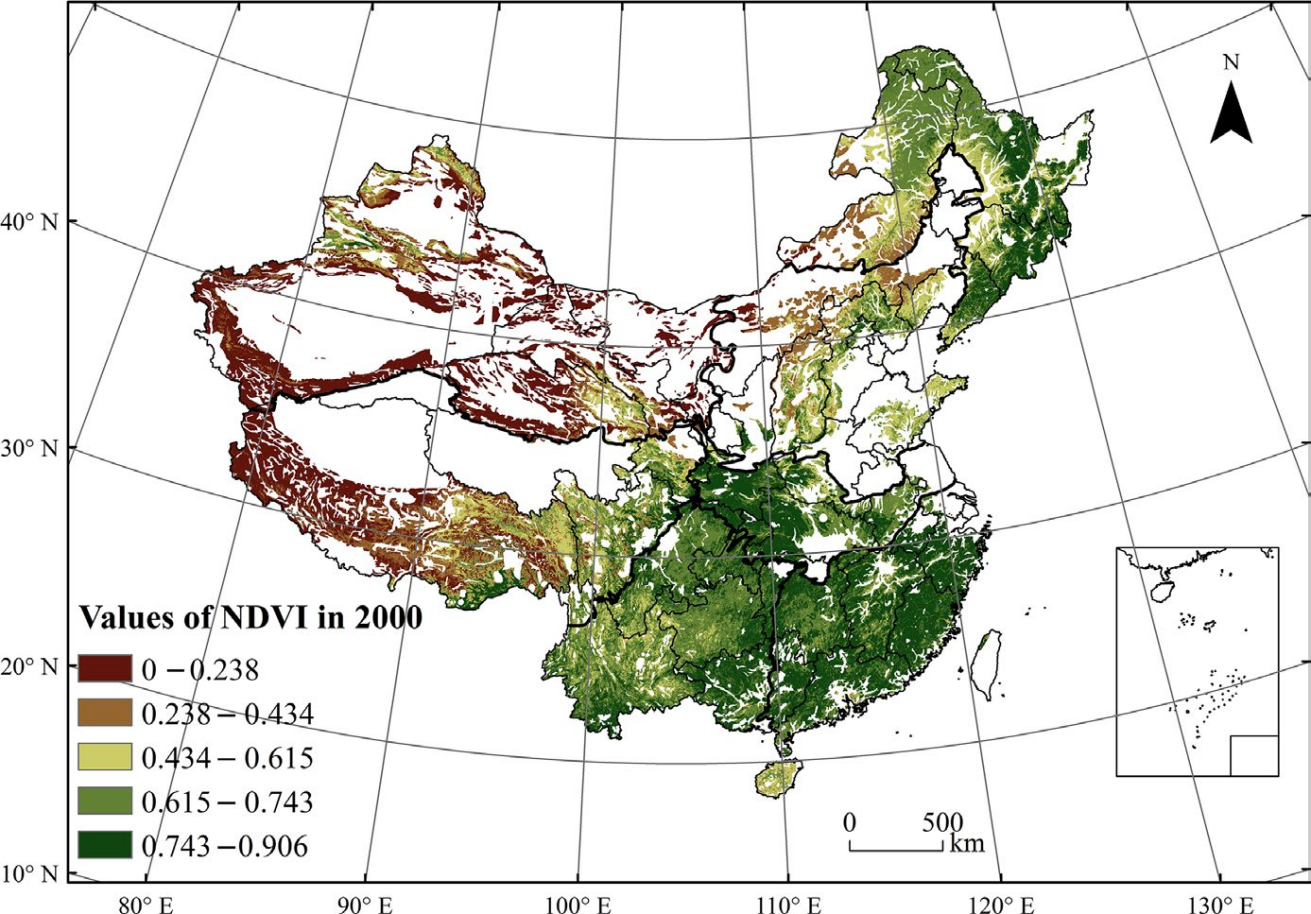
Spatial distribution of average NDVI values in the growing season in 2000 in mountainous areas of China

The NDVI values of China's mountainous areas in 2000 presented a gradually increasing trend from northwest to southeast (Figure 3). Near the Tarim Basin, the Taklamakan Desert and Western Tibet areas, the mean NDVI value was less than 0.2. In comparison, the highest vegetation coverage conditions were found in the southeast and central part, where most regions had a mean NDVI value greater than 0.615.

**Figure 4:**
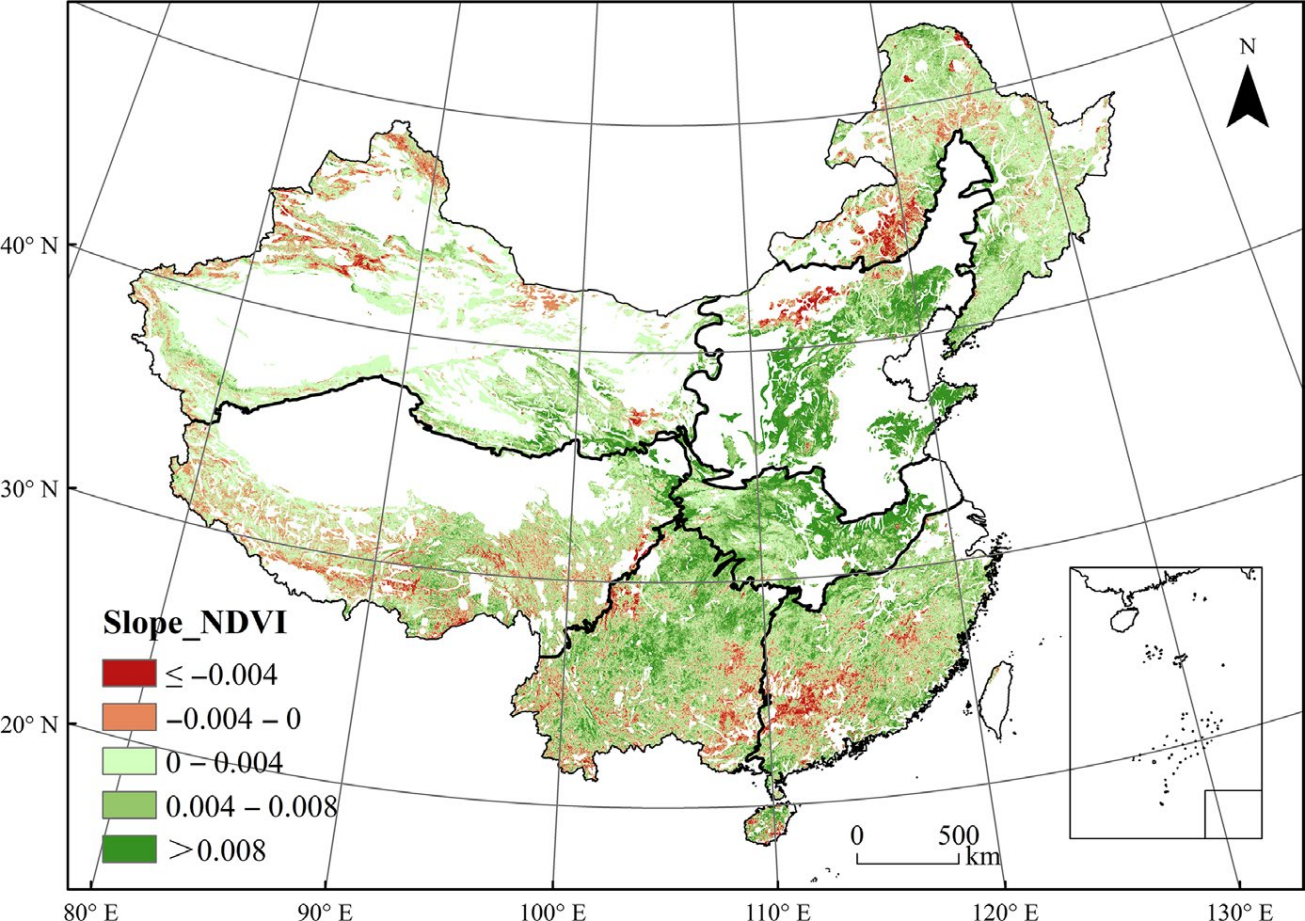
Spatial distribution of NDVI variation trends from 2000 to 2010 in mountainous areas of China

Overall, NDVI values during the period 2000 to 2010 showed an increasing trend, and the area of regions with increasing trend of NDVI accounted for 81.7% of the total study area; in northern China and central China, this increasing trend was especially obvious. However, in some of the mountainous areas in the northwest, northeast, Tibet, and south, NDVI values showed a decreasing trend (Figure 4).

### 4.2 Influences of population pressure change on vegetation greenness variation

Before model estimation, we adopted the Variance Inflation Factor (VIF) to carry out a full‐collinearity test on the explanatory variables. For all explanatory variables, VIF values were below 10, which means that there was no significant collinearity between variables.

Table 3 presents the influences of population pressure change on vegetation change at the national scale in mountainous areas of China. The explained variable is the variation trend of NDVI, and only the population pressure change was introduced into Model 1 as explanatory variable. At the significance level of 1%, population pressure change significantly affected the explained variable. With the decrease of population pressure, vegetation greenness showed tendency to increase. On the basis of Model 1, we successively introduced the land‐use intensity, climatic factors, and topographic factors into the model, with the results that population pressure, temperature, precipitation, gradient slope, aspect, and elevation all significantly influenced vegetation change at the national scale. In Model 4, although the introduction of climatic and topographic factors led to the decrease of the absolute value of the population pressure coefficient, population pressure had obvious influences on the dependent variable, which was still significant at the significance level of 1%. Using the stepwise regression method, the result that population pressure changes impact trend variation of vegetation greenness presented a robust consequence.

**Table 3:** Models of impact of population pressure change on NDVI slope at the national level in mountainous areas in China

Explained variable: Slope NDVI	Model 1	Model 2	Model 3	Model 4	Standardized coefficients of Model 4
Explanatory variable					
Population pressure change	−0.167*** (−5.00)	−0.167*** (−4.99)	−0.166*** (−4.92)	−0.106*** (−3.42)	−0.089
	[−0.167]	[−0.167]	[−0.166]	[−0.106]	
Control variables					
Land‐use intensity change		0.209 (0.63)	0.205 (0.62)	0.024 (0.08)	0.001
Climatic factors					
Slope temperature			0.019** (2.04)	0.047*** (5.19)	0.060
Slope precipitation			−0.004** (−2.15)	0.012*** (5.74)	0.136
Landform factors					
Average aspect				0.031* (1.64)	0.015
Average gradient				−0.170*** (−3.23)	−0.038
Average elevation				−9.544*** (−13.31)	−0.320
Dummy variables					
North	Yes	Yes	Yes	Yes	
Northwest	Yes	Yes	Yes	Yes	
Central	Yes	Yes	Yes	Yes	
Southwest	Yes	Yes	Yes	Yes	
Southeast	Yes	Yes	Yes	Yes	
Tibet	Yes	Yes	Yes	Yes	
Constant	23.33*** (38.38)	23.31*** (38.25)	23.89*** (38.05)	78.87*** (14.57)	
Number of observations	9,753	9,753	9,753	9,753	
Adjusted *R* ^2^	0.226	0.226	0.227	0.255	
AIC	93,681.09	93,682.19	93,677.11	93,312.42	
*F*	310.42	271.91	243.76	248.46	

The figures in [] are marginal effects of population pressure change; the figures in () are *t* values; *, **, *** are coefficients different from zero at 10%, 5%, and 1% significance levels, respectively; Region dummies = Yes. Standard error adjusted for 9,753 clusters in each sample.

The standard partial regression coefficient can reflect the influencing degree of the explanatory variables on the explained variables under the condition of controlling other variables (Cao et al., 2014). In Model 4 (Table 3), the standard partial regression coefficient shows that the main factors influencing the NDVI change, according to their influencing degrees from high to low, include average elevation, trend of precipitation variation, population pressure change, trend of temperature variation, gradient slope, and aspect.

To reveal the regional differences, we carried out further analysis on the factors that influence vegetation greenness change in Models 5–11. The results show that overall, population pressure change significantly influences the trend of the NDVI variation for the six regional models, except for the northwest. Among these models, the influence of population pressure on vegetation greenness was significant at the 1% significance level for Models 5, 8, 9, 10, and 11, and significant at the 5% significance level for Model 6. As introduced in Table 4, the marginal effects of the change of independent variables were presented. Thus, when population pressure decreased by one unit, in the case of controlling the other variables, the trend of NDVI variation increased by 9.7% in the northeastern mountainous areas of China (Table 4); in the northern and central mountainous areas, the trend of NDVI variation increased by 10.6% and 7.5%, respectively; while in the southeastern and southwestern areas, the trend of NDVI variation increased by 15.5% and 16.4% (Table 4). However, the only exception was the region of Tibet, where the population pressure had a positive impact on vegetation greenness change. The situation in Tibet was totally different, that is, a population increase by one unit could increase the trend of NDVI variation by 18.5%.

**Table 4:** Explanatory model for changes in vegetation greenness at different regions in mountainous areas of China

Explained variable: Slope NDVI	Model 5 Northeast	Model 6 North	Model 7 Northwest	Model 8 Central	Model 9 Southwest	Model 10 Southeast	Model 11 Tibet
Explanatory variable							
Population pressure change	−0.097*** (−2.70)	−0.106** (−2.07)	−0.205 (−1.55)	−0.075*** (−3.00)	−0.164*** (−6.45)	−0.155*** (−8.45)	0.185*** (6.92)
	[−0.097]	[−0.106]	[−0.205]	[−0.075]	[−0.164]	[−0.155]	[0.185]
Control variables							
Land‐use intensity change	0.002 (0.01)	−0.796 (−0.47)	1.796** (2.04)	−0.682 (−0.77)	−0.212 (−0.38)	−0.518 (−0.77)	−3.588** (−2.24)
Slope temperature	−0.187*** (−6.97)	0.149*** (2.88)	0.246*** (8.07)	−0.214*** (−3.84)	0.165*** (4.83)	0.065*** (2.84)	0.019* (1.65)
Slope precipitation	0.065 (1.62)	−0.139*** (−3.44)	0.011** (2.15)	−0.178*** (−9.53)	0.004 (0.39)	0.175*** (4.97)	−0.023*** (−4.44)
Average aspect	0.066* (1.74)	0.010 (0.09)	−0.035 (−0.88)	0.037 (0.54)	0.127*** (2.64)	0.036 (0.66)	0.044 (1.57)
Average gradient	−0.088 (−0.59)	2.183*** (6.96)	−0.846*** (−7.89)	−1.731*** (−6.89)	−1.132*** (−7.04)	−0.697*** (−3.27)	0.160* (1.81)
Average elevation	−10.518*** (−5.62)	−16.443*** (−4.16)	9.071*** (2.69)	8.426*** (4.20)	6.652*** (3.32)	−17.462*** (−10.27)	7.796 (1.58)
Constant	71.251*** (5.98)	169.068*** (5.89)	−43.820* (−1.74)	48.321*** (3.30)	−26.004* (−1.75)	121.282*** (9.78)	−36.577 (−0.97)
Number of observations	1,746	714	1,251	778	1,909	1,674	1,585
Adjusted *R* ^2^	0.113	0.217	0.153	0.374	0.084	0.242	0.085
*F*	36.39	30.54	41.19	82.31	25.32	69.04	26.80

The figures in [] are marginal effects of population pressure change; the figures in () are *t* values; *, **, *** are coefficients different from zero at 10%, 5%, and 1% significance levels, respectively. Standard error was adjusted for clusters in each sample; the results are robust.

Furthermore, Table 4 shows that there were large regional differences for the influences of land‐use intensity on the explained variables. In the northwestern and the Tibet areas, land‐use intensity had a significant positive influence on vegetation greenness change at the significance level of 5%. In other regions, land‐use intensity change had no significant impact on vegetation greenness change.

**Figure 5:**
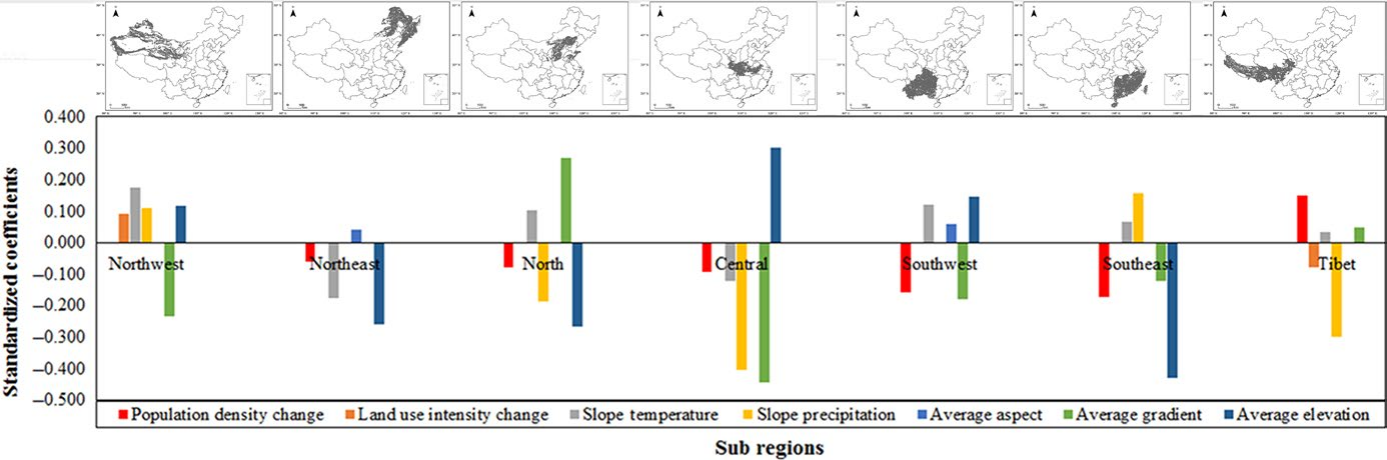
Standardized coefficients of significant explanatory variables based on multivariate linear regression models shown in Table 4 in subregions

Figure 5 shows the degree and direction of each significantly influencing factor on vegetation condition change in each subregion. According to the absolute value of the standardized coefficient of all the significant explanatory variables, the variable of population pressure change among all the variables which impact NDVI slope significantly in the southeastern, the southwestern, and the Tibet regions rank a lot higher than those in the northeastern, the northern, and the central regions.

Similar to most of the existing research results, temperature and precipitation positively influenced vegetation greenness in the national scale. However, the influences varied across regions. As this was not the focus of this study, we will further elaborate on this in the Appendix S1.

## 5 DISCUSSION

In this study, on the basis of analyzing the variation trend of the vegetation index in China's mountainous areas from 2000 to 2010, a variety of variables were selected, including natural and anthropogenic factors, to evaluate the factors influencing vegetation greenness change. First, we carried out rasterization on all explanatory variables, including temperature, precipitation, gradient slope, aspect, elevation, population pressure, and land use. Then, we quantitatively analyzed the influences of population pressure change and land‐use intensity change on vegetation greenness change.

**Figure 6:**
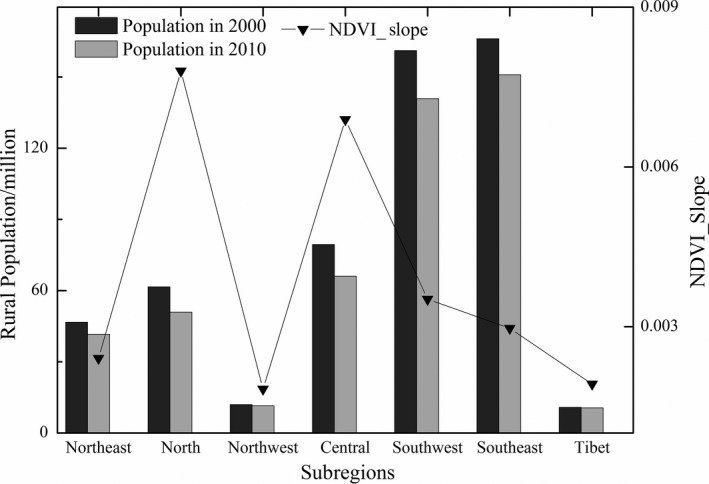
The rural population and average values of trend of NDVI variation in different areas

From the beginning of the 21st century, the rural population in China's mountainous areas has been decreasing significantly. In general, population pressure in two‐thirds of China's mountainous areas has been decreasing in the past 10 years. For instance, the northern, central, and southeastern mountainous areas presented the most obvious decrease. From 2000 to 2010, the rural populations of these three regions fell by 17.2%, 16.8% and 12.6%, respectively. Accordingly, the slope of NDVI change in these regions was relatively large, and vegetation greenness increase was significant (Figure 6). In contrast, in the northwestern and Tibet areas, the rural population only decreased by 4.1% and 1.8%, respectively, which was significantly lower than the national average of 17% (Li, Sun, Tan & Li, 2016). As a result, the NDVI increased slowly in this area.

With a decrease in rural population, vegetation index generally showed an increasing trend, except for a few regions which showed a decreasing trend and accounted for 18.3% of the total study area during the study period. Some scholars in China have come to similar conclusions (Cai, Yang, Wang & Xiao 2014; Han & Xu, 2008; Li, Sun, Tan & Li, 2016). For instance, Lu et al. (2015) have stated that China experienced both vegetation restoration and degradation with great spatial heterogeneity. In addition, Han and Xu (2008), using the correlation analysis method, found that demographic factors significantly affected vegetation productivity in the undeveloped regions with a great distance to the center of Chongqing, especially in the mountainous areas. In other countries in the developing world, research led to similar conclusions. For example, Olsson, Eklundh, and Ardö (2005) have found that the population emigration in marginal areas of the southern Sahara region increased the cultivated land abandonment rate, thus promoting the spontaneous recovery of vegetation to a certain degree. The results of a similar study have shown that cultivated land abandonment in mountainous areas caused by rural‐to‐urban labor migrants in St. Lucia, West Indies, had a certain facilitating effect on forest restoration in mountainous areas (Bradley, 2016). Based on these studies, it can be concluded that population pressure decrease positively impacts vegetation greenness change.

However, as mentioned in the introduction, previous researches have rarely analyzed the effects of human activity changes on vegetation greenness while controlling natural factors. In this study, we quantitatively analyzed the influences of human activities on the basis of controlling climatic and landform factors (Table 1). Based on this, we have come to two interesting conclusions. First, at the scale of 10 years, population decrease in China's mountainous areas had a significant influence on vegetation greenness change. Second, the marginal effect of population decrease to vegetation greenness change presented obvious differences from north to south. In the central area, on the premise of controlling other factors, a population decrease by one unit could increase the trend of NDVI change by only 7.5%, while the proportion was 16.4% in the southwestern area.

Nevertheless, in the mountainous areas of Tibet, the impact of population pressure change on vegetation greenness change turned out to be positive, which is totally different from the results in the other researched areas. During the first ten years of the 21st century, the rural population did not decrease dramatically in Tibet's mountainous areas. On the contrary, in most of the researched areas of Tibet, the rural population, according to the census data, increased due to low rural–urban migration and a high birth rate. In these areas where populations were centered, grassland was artificially irrigated which contributed to the improvement of vegetation conditions. Studies showed that through irrigation, the grassland biomass in Tibet could be significantly increased (Ganjurjav et al., 2014, 2015). Above all, the proportion of shrubs and broad‐leaved forbs was also increased under irrigation conditions.

Generally speaking, with higher land‐use intensities and human activities, vegetation greenness decreases. However, in this study, land‐use intensity had no significant impact on the dependent variables in most of the models. This may mainly be related to the definition of land‐use intensity in this study. We calculated land‐use intensity according to equation (2), which divides land‐use status into four different grades. Nevertheless, this discontinuity of variables cannot fully reflect the influences of land use and masks a large amount of vegetation responses to land‐use change.

Remarkably, in Model 7 and 11 (Table 4), land‐use intensity has a significant positive influence on the dependent variables; the increase of land‐use intensity can promote vegetation greenness. This situation occurs in northwestern China and is, most likely, mainly related to the development of irrigated agriculture in the area. In the valley and piedmont zones of the northwestern area, the temperature rise in recent years has increased the amount of alpine snow water, which promoted the development of irrigated agriculture in these regions, thus improving regional vegetation (Ta, Dong, & Caidan, 2006). Previous studies have shown that from 1975 to 2005, the large‐scale development of cultivated land in Xinjiang has significantly influenced regional vegetation (Wang, Wang, Zhang, & Duan, 2014). The vigorous development of irrigated agriculture in these regions has improved the vegetation conditions to some extent.

In addition, vegetation change is affected not only by natural conditions and human factors, but also by other factors such as land use policy, related projects, and policies of vegetation protection (Li, Wu & Huang, 2012; Lu et al., 2015; Luck, Smallbone, & O'brien, 2009). Especially since the 1990s, large‐scale ecological protection and afforestation projects have been significantly affecting vegetation restoration (Lu, Fu, Wei, Yu, & Sun, 2011). The variable of land‐use intensity in this study can, to a certain extent, reflect the influence of the “grain‐to‐green” policy. However, land‐use changes do not fully reflect the influence of policies on vegetation greenness. Further studies are therefore required to assess the impacts of population change on vegetation greenness change.

## AUTHORS CONTRIBUTION

Minghong Tan and Wei Li designed the research. Wei Li, Minghong Tan, and Yahui Wang performed data collection and analysis. Wei Li and Minghong Tan interpreted the results and drafted the manuscript. All authors contributed to discussion and writing. Xiubin Li and Minghong Tan revised the early version.

## CONFLICT OF INTEREST

None declared.
