# Supplementary material for: Influences of population pressure change on vegetation greenness in China's mountainous areas
Source: Ecol Evol. 2017 Sep 25;7(21):9041–53. doi: 10.1002/ece3.3424 (PMC5677483; doi:10.1002/ece3.3424)
Supplement: Supplementary file 1 [file ECE3-7-9041-s001.docx]

# Supporting Information:

Similar to most existing research results, temperature and precipitation positively significantly influenced vegetation greenness in the national scale. However, the influences varied greatly across regions. For instance, in the Northeast region, temperature had the strongest negative effect, and precipitation had a positive effect on the trend of NDVI variation. This is probably caused by the arid climate in this region. Zhao (2013) investigated that annual mean temperature increased at a significant rate of 0.35°C per decade while rainy days had a significant decreasing trend during 1961-2010 in northeastern China. Such climate causes severe water stress for vegetation in years with low precipitation and high temperature.

In northern and central regions, unlike what we had expected, precipitation had negative effects on vegetation greenness. It may be caused by different reasons in different regions. Taking the Northern region as an example, the precipitation showed an increasing trend in the northern part of this region, while NDVI presented a decreasing trend (Fig S1*). Here, precipitation might not be the main factor affecting vegetation greenness. It is related to the reclamation of cultivated land, mowing, and grazing land-use types in the eastern Inner Mongolia (Gong *et al*, 2014).

**Figure** **S1*. The trend of NDVI variation and the trend of precipitation variation in the Northern mountainous area**


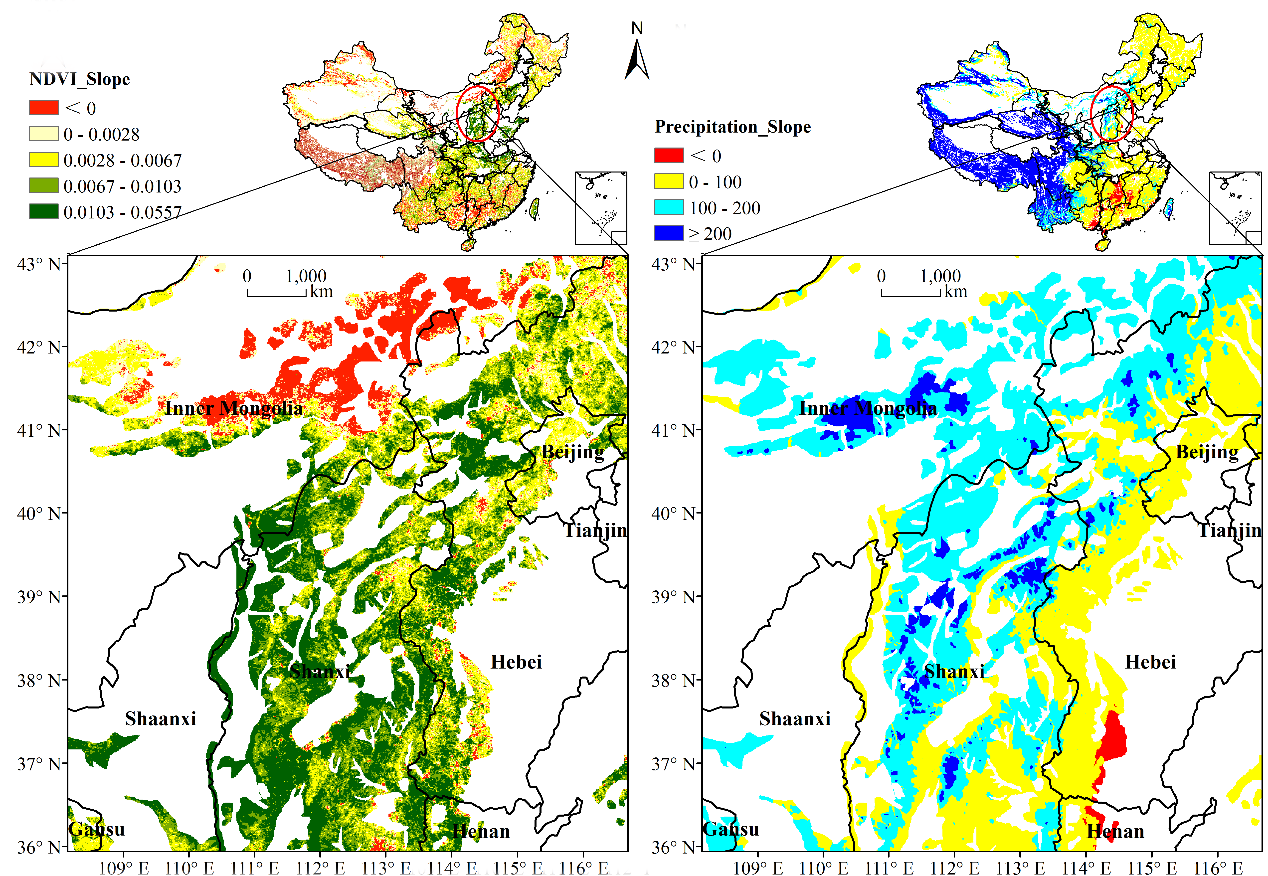


Reference:

Gong J-R, Wang Y, Liu M, Huang Y, Yan X, Zhang Z & Zhang W (2014) Effects of land use on soil respiration in the temperate steppe of Inner Mongolia, China. *Soil and Tillage Research*, **144**, 20-31. doi: 10.1016/j.still.2014.06.002

Zhao CY, Wang Y, Zhou XY, Cui Y, Liu YL, Shi DM, Yu HM, Liu YY. (2013) Change in climatic factors and extreme climate events in northeast China during 1961-2010. *Advances in Climate Change Research*, **4**(2): 92-102. doi: 10.3724/SP.J.1248.2013.092
